# Floristic composition in ecotone forests in northern Brazilian Amazonia: preliminary data

**DOI:** 10.3897/BDJ.7.e47025

**Published:** 2019-10-29

**Authors:** Williamar Rodrigues Silva, Carlos Darwin Angulo Villacorta, Ricardo Oliveira Perdiz, Hugo Leonardo S. Farias, Andressa Sampaio Oliveira, Arthur Camurça Citó, Lidiany Camila Silva Carvalho, Reinaldo Imbrozio Barbosa

**Affiliations:** 1 UFRR/PRONAT, Boa Vista-RR, Brazil UFRR/PRONAT Boa Vista-RR Brazil; 2 Centro de Investigación, Enseñanza y Producción Agroforestal - CEPIAGRY, Yurimaguas, Peru Centro de Investigación, Enseñanza y Producción Agroforestal - CEPIAGRY Yurimaguas Peru; 3 INPA/PPGBOT, Manaus-AM, Brazil INPA/PPGBOT Manaus-AM Brazil; 4 INPA/NAPRR, Boa Vista-RR, Brazil INPA/NAPRR Boa Vista-RR Brazil; 5 University of Exeter, Exeter, United Kingdom University of Exeter Exeter United Kingdom

**Keywords:** Amazon, Brazil, forest inventory, Maracá Island, palms, Roraima, tree species

## Abstract

**Background:**

Ecotone has been defined as “*a multi-dimensional environmentally stochastic interaction zone between ecological systems with characteristics defined in space and time, and by the strength of the interaction*” (Hufkens et al. 2009). This is a known concept to define transitional zones between two or more ecological communities, ecosystems or biotic regions. Ecotone forests, dispersed in northern Brazilian Amazonia, are natural formations which have been largely affected by anthropogenic impacts, such as deforestation and fire. Maracá Ecological Station, State of Roraima, Brazil, is a protected area with extensive representations of ecotone forests in this region of the Amazonia. Forest inventories and floristic surveys are important as they extend our knowledge (1) of forest structure and tree species composition and (2) of tree and palm species ecology in this region of the Amazonia. Both improve our ability to predict changes in plant diversity, considering the future scenarios of climate change in comparison with previous surveys performed in Maracá.

**New information:**

We present a forest inventory carried out in 129 plots (10 m x 50 m; 6.45 ha in total) dispersed in a grid (5 km x 5 km) located in a forest zone ecotone in the eastern part of Maracá Ecological Station. All stems (tree + palm) with diameter at breast height ≥ 10 cm were recorded, identified and measured. A total of 3040 stems were recorded (tree = 2815; palm = 225), corresponding to 42 botanic families and 140 identified species. Seven families and 20 genera contained unidentified taxa (12.2%). Sapotaceae (735 stems; 10 species), Leguminosae (409; 24) and Rubiaceae (289; 12) were the most abundant families. *Peltogyne gracilipes* Ducke (Leguminosae), *Pradosia surinamensis* (Eyma) T.D.Penn. (Sapotaceae) and *Ecclinusa guianensis* Eyma (Sapotaceae) were the species with the highest importance value index (~ 25%). The dominance (m^2^ ha^-1^) of these species corresponds to > 36% of the total value observed in the forest inventory. Our dataset provides complementary floristic and structure information on tree and palm in Maracá, improving our knowledge of this Amazonian ecotone forest.

## Introduction

The Pan-Amazon or Continental Amazon has the largest tropical forest area on the planet with > 6 million km^2^ (MapBiomas 2019, WWF 2019). Estimates based on > 1,100 permanent plots scattered throughout the region indicate that the tree richness ranges between 7-10,000 species occupying a great diversity of habitats (Cardoso et al. 2017, ter Steege et al. 2019). Brazilian Amazonia accounts for the largest physical area of this region (Salati and Vose 1984; > 5 x 10^6^ km^2^). However, it has been long threatened by a series of anthropogenic impacts, such as the replacement of native forest by pasture and soybean planting (Almeida et al. 2016, Fearnside 2006), combined with an increase in burned areas of primary and secondary forests (Alencar et al. 2015, Aragão et al. 2018, Barni et al. 2015). Modern anthropogenic activities, associated with global warming, have a negative effect on tree diversity and reduce the conservation status of Amazonian forests (Barlow et al. 2016, Esquivel-Muelbert et al. 2019). Despite having large areas of ombrophilous forests, the ecotone forests are important ecological areas because they occupy the peripheral zone to the Amazon basin (Central Amazonia) and are considered as the forest formations most impacted by anthropogenic activities in the Southern (Marques et al. 2019, Nogueira et al. 2015) and Northern (Barni et al. 2015, Santos et al. 2013) Amazonian "arcs of deforestation". The accelerated increase of anthropogenic activities within the Amazonian ecotones has been a major contributor to the fact that the region as a whole is now approaching to the "tipping point", limiting forest growth and potentially favouring low-density environments when compared to those currently supported by the region (Lovejoy and Nobre 2018).

Several floristic surveys and forest inventories have been carried out in these peripheral regions, especially from 1980-1990 (Nelson and Oliveira 2001). However, this period was insufficient to accumulate realistic information on forest structure and floristic in view of the continuous advance of anthropogenic activities. This impedes the reliable characterisation of plant diversity in these peripheral Amazonian regions. One such area is the State of Roraima, located in the northern part of the Brazilian Amazonia. This ~225,000 km^2^ area contains ecotone forest zones of great geoecological importance because they are located between Guiana Shield (highland savannas and tropical dry forests) and Central Amazonia (lowland tropical forests) (Barbosa and Bacelar-Lima 2008, Oliveira et al. 2017), which gives them a unique phytogeographical and ecoevolutionary history, where a high degree of endemism is observed and unique vegetation sets exist (Nascimento and Proctor 1997, Milliken and Ratter 1998). However, the region has received few forest inventories and floristic surveys (Suppl. material 1); main of them are associated with the monodominant forests (Nascimento and Proctor 1997, Nascimento et al. 1997), campinas and campinaranas (Barbosa and Ferreira 2004, Damasco et al. 2013) and forest fragments dispersed in savannas (Sette-Silva 1993, Santos et al. 2013, Jaramillo 2015).

In an attempt to expand studies on plant diversity in this part of the Amazon, two PPBio (Biodiversity Research Program, https://ppbio.inpa.gov.br) 25-km^2^ research grids were installed in areas defined as ecotone forests in Roraima taking into account the Brazilian Vegetation Classification System (Brazil-IBGE 2012): Maracá Ecological Station and Viruá National Park. Both are protected areas under Brazilian government management. Surveys of structure (vertical and horizontal) and tree species composition of the main forest types of these research grids have been expanded in Maracá (Nascimento et al. 2014, Nascimento et al. 2017, Villacorta 2017) and Viruá (Damasco et al. 2013, Barbosa et al. 2017). Accordingly, the current study provides preliminary data from a forest inventory carried out in the Maracá research grid. These data expand the scale of floristic and structural observations in this northern Amazonian ecotone zone from those initiated by Milliken and Ratter (1998) and Nascimento et al. (1997).

## Project description

### Title

"*Tree growth and mortality in Roraima ecotonal forests*" (Crescimento e mortalidade de árvores em florestas ecotonais de Roraima). The dataset is associated with the PhD thesis of Williamar Rodrigues Silva (Analysis of environmental conditions and climate variability on tree growth and mortality in ecotonal forests of Maracá Island, Roraima), PRONAT/UFRR, Boa Vista, Roraima, Brazil.

### Personnel

Williamar Rodrigues Silva

### Study area description

**Figure 1.**
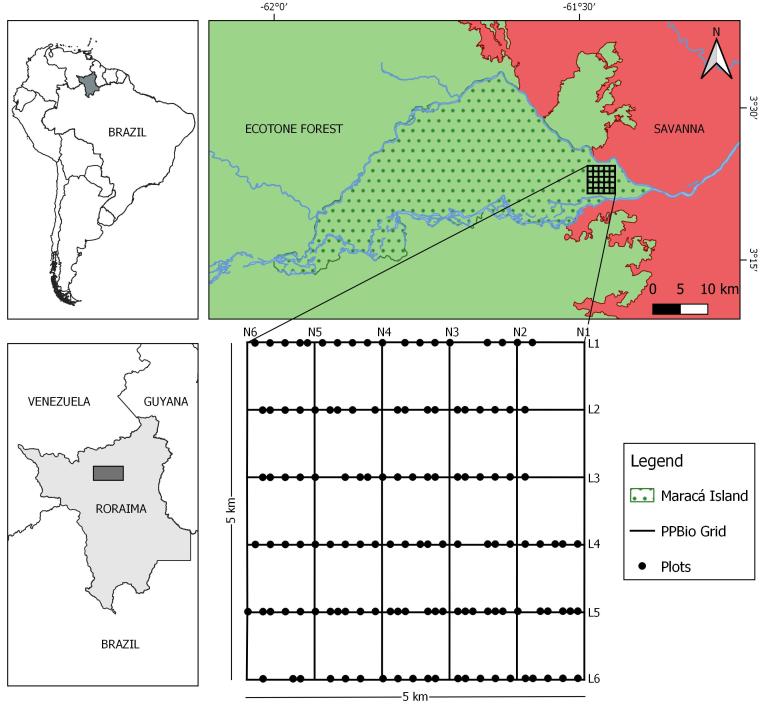
Study area with emphasis on the PPBio research grid (Maracá), Roraima, northern Brazilian Amazonia.

The dataset was constructed from a forest inventory conducted in the ecotone forests of eastern Maracá Island, state of Roraima, northern Brazilian Amazonia: 3.360086 N to 3.405148 N / -61.44169 W to -61.48583 W (Fig. 1 ; figure was constructed using QGIS 2019 free software). A mosaic of ombrophilous and seasonal (deciduous and semideciduous) forest types predominates in this region. Maracá Island is part of the Maracá Ecological Station, a Brazilian protected area (~ 101,000 ha), located between two channels of the Rio Uraricoera (Maracá and Santa Rosa). The study region is a continuum of ecotone forests that have contact with the largest savanna area in northern Brazilian Amazonia (Barbosa et al. 2007, Barbosa and Campos 2011). Although it was inhabited until the mid-1970s (Proctor and Miller 1998) and there has been an increase in anthropogenic pressures in adjacent regions (Couto-Santos et al. 2014), the conservation status of Maracá remains excellent and the area may be considered an important representative of mature forest tree species diversity for the region. Eastern Maracá soil classes are directly related to drainage and relief (51-99 m a.s.l.). These local constraints determine different forest types that occupy such seasonal flooding-free areas as moist lowlands and rocky slopes (Villacorta 2017). Well-drained soils are situated in areas of higher reliefs (Ultisols) or on slopes dominated by base-rich rocky soils. Soils occupying the lower-lying areas are poorly drained with a dominance of typical hydromorphic sandy soils (Nortcliff and Robison 1998). Regional climate is characterised as a transition between Aw/Am climate subtypes by the Köppen classification (Barbosa 1997). Average annual rainfall is ~ 1900 mm (1986-2010), with a rainy season between April and September (140-420 mm month^-1^) and a dry season between October and March (40-130 mm month^-1^) (Couto-Santos et al. 2014).

### Funding

CNPq (Proc. CNPq 403591/2016-3) funded data collection.

## Sampling methods

### Study extent

The floristic survey was carried in March and December 2017.

### Sampling description

The PPBio research grid was installed between 2005-2006 in eastern Maracá Island (see details in https://ppbio.inpa.gov.br/sitios/maraca/infra). The grid is formed by 12 x 5-km trails (six parallel trails in N-S direction and six in E-W direction, forming a 1-km resolution grid (Magnusson et al. 2005, Pezzini et al. 2012). A floristic survey was conducted within the grid in two periods (March and December 2017) to sample tree and palm species richness and composition in the ecotone forest mosaic. The floristic survey was carried out in 129 plots (10 m x 50 m each one; 6.45 ha in total), distributed on the six E-W trails (Fig. 1; Suppl. material 2). Each plot was systematically arranged along each trail with a minimum distance of 150 m between them to avoid pseudoreplication. This method was adopted to better cover the great environmental heterogeneity, characteristic of ecotone forests. The floristic survey included measurements of stem diameter (diameter tape - precision 0.1 cm), collecting botanical material and general description of the individuals/species, following standardised tree measurement methods established for PPBio grids and modules (Castilho et al. 2004). Palm height was estimated with Android technology using the Measure Height App (Oü 2014), while trees height was estimated by regression using a Maracá-specific allometric model (Barbosa et al. 2019).

**Analysis**: We calculated abundance (number of stems) and richness (number of species) for all arboreal stems ≥ 10 cm in diameter (tree and palm) recorded in the floristic inventory. Frequency and dominance (absolute and relative) were also calculated to estimate the importance value indexes for family (FIV) and species (IVI). All stems were classified by diameter size and total height classes to analyse the horizontal-vertical structure. We tabulated all floristic composition and diameter data (n = 129 plots) using a multiple interacting spreadsheet programme (Excel Office 365) and applied the vegetation analysis methods described in Kent and Coker (1994) to calculate frequency, dominance and importance value indexes (family and species).

### Quality control

All observed individuals were collected and morphotyped in the field, then subsequently botanically identified. Vouchers representing most of the inventoried species were deposited in the herbarium of the INPA (Instituto Nacional de Pesquisa da Amazônia), MIRR (Museu Integrado de Roraima) and UFRR (Universidade Federal de Roraima). The species identification was performed by Antônio Tavares Mello by comparison with exsiccates in the INPA Herbarium and by R.O. Perdiz and R. I. Barbosa, taking into account comparison with exsiccates in the INPA, UFRR and MIRR herbariums and material available via the digitised collection of project REFLORA (Reflora - Virtual Herbarium, available at http://reflora.jbrj.gov.br/reflora/herbarioVirtual/). Species scientific names were checked and corrected by comparison with data from Brazil Flora Group (BFG 2015). Family-level delineations followed APG-IV (2016).

### Step description

The floristic survey described here was done in two periods (March and December 2017).

## Geographic coverage

### Description

Data was collected in 129 plots across PPBio-Maracá research grid located on the eastern end of Maracá Ecological Station (see Fig. 1). All permanent plots are located on coordinates: 3.40515 N and -61.48583 W; 3.36009 N and -61.44169 W (Datum WGS 84).

## Taxonomic coverage

### Description

We observed a total of 3040 stems (tree = 2815; palm = 225) in the floristic inventory (129 plots = 6.45 ha), corresponding to 42 families, 119 genera and 140 identified species (Table 1). Seven families and 20 genera contained unidentified taxa (12.2% of total), all of which occurred due to a lack of appropriate taxonomic material (individuals dormant or without fertile material) to provide a definite determination. Sapotaceae (735 stems; 10 species), Leguminosae (409; 24) and Rubiaceae (289; 12) were the most important families with the highest family importance values (FIV = 42.4%). *P. gracilipes* Ducke (Leguminosae), *P. surinamensis* (Eyma) T.D.Penn. (Sapotaceae) and *E. guianensis* Eyma (Sapotaceae) were the species with the highest importance value index; ~ 25% in total (Table 2). Dominance (m^2^ ha^-1^) of these species corresponds to > 36% of the total observed in the forest inventory.

Palm stems (225) were observed only amongst the 10-40 cm diameter size classes, with the main distribution concentrated in the 20-30 cm class (62.2%) (Table 3). Most tree stems fell within the 10-20 cm class (56.7%), with the largest diameter class (> 50 cm) representing 5.9% of measured stems. The species with the largest structural parameters were *Laetia procera* – Salicaceae (stem diameter = 89.3 cm; total height = 33.5 m; stems = 1), *Pochota fendleri* (Seem) W.S. Alverson & M.C. Duarte – Malvaceae (63.4 cm; 25.6 m; 9) and *Anacardium giganteum* Hancock ex Engl. - Anacardiaceae (63.3 cm; 30.2 m; 2), all of these being encountered at low abundance. Thirty-four species were represented by a single stem.

## Usage rights

### Use license

Creative Commons Public Domain Waiver (CC-Zero)

### IP rights notes

These data can be freely used, provided their source is cited.

## Data resources

### Data package title

Tree species composition in ecotone forests of the eastern Maracá Island, Roraima, northern Brazilian Amazonia: preliminary data

### Resource link

https://ipt.sibbr.gov.br/sibbr/resource?r=maraca_comp_floristic

### Alternative identifiers

https://www.gbif.org/dataset/01d28467-87a1-4d64-ba40-4e3e1cc9091b

### Number of data sets

1

### Data set 1.

#### Data set name

Composição de espécies arbóreas em florestas de ecótono do leste da Ilha de Maracá, Roraima, norte da Amazônia brasileira: dados preliminares (Tree species composition in ecotone forests of the eastern Maracá Island, Roraima, northern Brazilian Amazonia: preliminary data).

#### Data format

Darwin Core Archive DwC-A

#### Number of columns

24

#### Description

Occurrence of tree and palm species identified during a floristic survey in 129 plots installed in Maracá Island, Roraima, northern Brazilian Amazonia. Dataset consist in the occurrence.txt file containing the DwC-Attributes.

**Data set 1.** 

Column label	Column description
basisOfRecord	The specific nature of the data record.
language	Language of the resource.
institutionCode	Institution that has custody of the object or information about its registration.
collectionCode	The name or acronym of the collection or dataset from which the record is derived.
occurenceID	A identifier for the occurrence (unique).
catalogNumber	An identifier (preferably unique) for the record within the dataset or collection.
habitat	Description of the habitat in which the event occurred.
continent	The Continent of the occurrence.
country	The Country of the occurrence.
stateProvince	The State or Province of the occurrence.
county	The County of the occurrence.
locality	The location-specific description.
decimalLatitude	The geographical latitude in decimal degrees of the geographical centre of a location.
decimalLongitude	The geographical longitude in decimal degrees of the geographical centre of a location.
geodeticDatum	The ellipsoid, geodetic datum or spatial reference system (SRS) in which the geographical coordinates given in decimalLatitude and decimalLongitude are based.
kingdom	Full scientific name of the kingdom in which the taxon is classified.
family	Full scientific name of the family in which the taxon is classified.
genus	Full scientific name of the genus in which the taxon is classified.
specificEpithet	The name of the species-specific epithet.
intraspecificEpithet	The name of the terminal or lower-level infraspecific epithet of the scientific name.
scientificName	The full scientific name. It must be the name of lowest level taxonomic rank that was determined.
vernacularName	Common or vernacular name.
taxonRemarks	Comments or notes about the taxon or name.
identificationQualifier	A brief phrase or standard term ("cf.", "aff.") to express the determiner's doubts about identification.

## Additional information

### Discussion

Our floristic composition results for ecotone forests on eastern Maracá Island complement previous investigations carried out at the macro (Milliken and Ratter 1998) and micro (Thompson et al. 1992, Nascimento et al. 1997, Nascimento and Proctor 1997) sampling scale at this location. Although our study was not conducted to differentiate the forest types comprising this ecotone region, the broad dispersion of 129 small plots (0.05 ha each) over a wide area (25 km^2^), aids understanding of the various forest types present in the area. This occurred since small plots were better suited to the environmental variability scale of this ecotone region, because they covered specific sampling areas of each forest type comprising the ecotonal mosaic. Use of plots smaller than those commonly used in tropical forest inventories (0.5-1.0 ha; e.g. Phillips et al. 2016) may be an alternative for floristic surveys or forest inventories in regions with high environmental variability. However, sampling, using small plots in Maracá, followed basic rules: (i) sampling design maintained the independence of each sampling unit, (ii) large number of samples (> 100) to adequately represent the environment and (iii) annual tree censuses. All these recommendations are important to reduce the coefficient of variation between samples and to avoid temporal measurement problems associated with the descriptors of floristic composition, dynamics and forest structure (Keller et al. 2001, Wagner et al. 2010).

Our study recorded stem density (471 stems ha^-1^) as the basal area (26.3 m^2^ ha^-1^) values compatible with those of Thompson et al. (1992) (range 340-476 ind ha^-1^; 21.7-26.7 m^2^ ha^-1^) and Nascimento et al. (2014) (408-512 ind ha^-1^; 26.0-32.5 m^2^ ha^-1^), both studies also being performed in eastern Maracá, but using smaller sampling areas. Overall, our study indicates that the main families described in previous inventories, such as Arecaceae, Burseraceae, Chrysobalanaceae, Leguminosae, Rubiaceae and Sapotaceae (Milliken and Ratter 1998, Nascimento et al. 1997), retained the proportional representation they had in surveys two decades earlier – indicating the site had little anthropic impact in the intervening time period. Additionally, the similarity of our larger sample to smaller-scale efforts of the past indicates that compositional units repeat, underscoring the near-fractal nature of the vegetation mosaic. The main families observed in Maracá are common throughout the Amazon, are always present in forest inventories and floristic surveys and almost always have the largest number of species, so that they are considered to be hyperdominant in the region (ter Steege et al. 2013). For example, Leguminosae and Sapotaceae (Condé and Tonini 2013) and Burseraceae, Chrysobalanaceae and Leguminosae (Alarcón and Peixoto 2007) were also families with high importance value indices in other Roraima forest types. We emphasise that the importance of each family in these surveys differs from Maracá, indicating that the ecotone forests of this region have their own composition and dynamics, so differing from mosaics or forest types observed elsewhere in the State of Roraima.

As with the families, most plant species, described in past inventories, are also present in our list, especially those with higher IVI (*P. gracilipes*, *P. surinamensis*, *E. guianensis*), besides Lecythis corrugata subsp. rosea (Lecythidaceae), *Attalea maripa* (Arecaceae) and *Licania discolor* (Chrysobalanaceae), all of which were strongly represented in previous surveys. The case of *L. discolor* Pilg. is the most interesting because the individuals attributed to this species in our work were largely attributed to *L. kunthiana* Hook.f. in previous surveys, a very similar taxonomic species, but were far less abundant at Maracá. The proportion and spatial distribution of species inventoried in Maracá ecotone forests are conditioned by a variety of environmental filters. For example, *P. gracilipes* is a deciduous species endemic to this area of the northern Amazonia that can form monodominant agglomerations (Nascimento and Proctor 1997). However, the monodominance of this species occurs only in seasonally flooded areas in bottom lands, with high Fe^+2^ concentration in the soil (Nascimento et al. 2017). Such conditions are non-existent or rare in fertile and flood-free soils (Villacorta 2017). These distinct *P. gracilipes*-associated environmental characteristics reveal an ecotone region where forest types are intertwined with topographic, edaphic and hydrological constraints.

The species *P. gracilipes* plays an important ecological role (IVI = 10.4%) and it has been used to delimit forest types on Maracá. For example, Milliken and Ratter (1998) used this species to define forests monodominant with *Peltogyne* as “*Peltogyne gracilipes* forest”. Similarly, Nascimento et al. (1997) used this species to divide the Maracá ecotone zone into three forest types: (i) PRF (*Peltogyne*-rich Forest) or forests monodominate with *P. gracilipes*, (ii) FWP (Forest without *Peltogyne*) or types where this species do not occur and (iii) PPF (*Peltogyne*-poor Forest), which are mixed types with low *P. gracilipes* abundance. The three types correspond analogously to the Deciduous Seasonal Forest (= PRF), Semideciduous Seasonal Forest (= PPF) and Open Ombrophilous Forest (= FWP) of the Brazilian Vegetation Classification System (Brazil-IBGE 2012), all with distinct hydro-edaphic and topographic characteristics (Carvalho et al. 2018). These forest types definitions have enhanced the understanding of variation in biomass/carbon stocks estimates for the Maracá Island (Nascimento et al. 2007, Nascimento et al. 2014).

### Conclusion

The results of this study agree with data from previous investigations, indicating that the environmental heterogeneity of the ecotone forests of eastern Maracá Island influences floristic richness and structural distinctions, with *P. gracilipes* abundance acting as a descriptor variable for forest types. Consequently, the floristic survey conducted by this study is important because it expands our knowledge of forest structure and tree species composition in ecotone zones of the northern Brazilian Amazonia, improving our ability to predict changes in species composition and plant diversity when we take into account comparisons between previous forest inventories performed in Maracá. Finally, this study contributes to the local floristic knowledge, complements the herbarium collections with new collections, allows the development of similar studies and also enables the elaboration of management plans for the conservation of the local biota.

## Supplementary Material

D0A91D56-EB29-5E94-869A-75100CF97B40 10.3897/BDJ.7.e47025.suppl1 Supplementary material 1 The main studies in Roraima ecotone areas involving forest inventories and floristic surveys. Data type: List of studies
File: oo_348828.docx https://binary.pensoft.net/file/348828 Williamar Rodrigues Silva and Reinaldo Imbrozio Barbosa

E31CF98D-8C43-5489-B902-2E7FF087FF31 10.3897/BDJ.7.e47025.suppl2 Supplementary material 2 Geographic coordinates (degree - lat/long; DATUM WGS84) and altitude (m a.s.l.) of sampling units (plotID) in the eastern Maracá Island. Data type: geographic coordinates (lat/long)
File: oo_348829.txt https://binary.pensoft.net/file/348829 Williamar Rodrigues Silva, Carlos Darwin Angulo Villacorta and Reinaldo Imbrozio Barbosa

## Figures and Tables

**Table 1.** List of the most important arboreal families observed for eastern Maracá Island, where: Abundance = number of stems ≥ 10 cm in diameter; BA = total basal area (m^2^); ReAb = relative abundance (%); ReFq = relative frequency (%); ReDo = relative dominance (%); FIV = family importance value (%) representing the mean between ReAb, ReFq and ReDo. Raw data can be accessed on the GBIF website (Silva et al. 2019).

Family	Genus	Species	Abundance	AB m^2^	ReAb (%)	ReFq (%)	ReDo (%)	FIV (%)
Sapotaceae	4	10	735	4597.95	24.2	10.4	27.1	20.5
Leguminosae	18	24	409	3615.21	13.5	7.8	21.3	14.2
Rubiaceae	9	11	289	860.76	9.5	8.5	5.1	7.7
Arecaceae	6	6	225	1228.04	7.4	7.4	7.2	7.4
Lecythidaceae	5	6	239	880.84	7.9	7.2	5.2	6.7
Burseraceae	2	8	160	705.41	5.3	5.7	4.2	5.0
Chrysobalanaceae	7	7	116	831.58	3.8	5.1	4.9	4.6
Apocynaceae	2	4	114	402.16	3.8	4.9	2.4	3.7
Moraceae	7	8	91	344.19	3.0	4.5	2.0	3.2
Annonaceae	3	5	86	214.72	2.8	3.0	1.3	2.4
other 33	56	52	576	3286.97	18.9	35.5	19.4	24.6
Total	119	141	3040	16967.83	100	100	100	100

**Table 2.** List of the most important arboreal species (stem diameter ≥ 10 cm) observed on eastern Maracá Island, northern Brazilian Amazonia, where: Density = stem density (absolute and relative), Dominance = BA in m^2^ (absolute and relative). Frequency = number of plots where the species was observed (relative), IVI = importance value index (mean between %Density, %Dominance, %Frequency). The raw data can be accessed on the GBIF website (Silva et al. 2019).

Species	Family	Abundance (ind)	Density		Dominance		Frequency	IVI
			ind ha^-1^	%	m^2^ ha^-1^	%	%	
*Peltogyne gracilipes* Ducke	Leguminosae	299	46.4	9.84	4.7	17.92	3.39	10.38
*Pradosia surinamensis* (Eyma) T.D.Penn.	Sapotaceae	170	26.4	5.59	3.0	11.22	4.96	7.26
*Ecclinusa guianensis* Eyma	Sapotaceae	276	42.8	9.08	1.9	7.09	4.58	6.92
*Attalea maripa* (Aubl.) Mart.	Arecaceae	156	24.2	5.13	1.5	5.86	4.33	5.11
Lecythis corrugata subsp. rosea (Spruce ex O.Berg) S.A.Mori	Lecythidaceae	178	27.6	5.86	0.9	3.47	4.02	4.45
*Alseis longifolia* Ducke	Rubiaceae	160	24.8	5.26	0.9	3.56	3.58	4.13
*Pouteria surumuensis* Baehni	Sapotaceae	113	17.5	3.72	0.8	2.99	3.26	3.32
*Pouteria hispida* Eyma	Sapotaceae	85	13.2	2.80	1.0	3.73	3.01	3.18
*Protium stevensonii* (Standl.) Daly	Burseraceae	98	15.2	3.22	0.8	2.95	2.82	3.00
*Licania discolor* Pilg.	Chrysobalanaceae	72	11.2	2.37	1.0	3.80	2.70	2.96
*Himatanthus articulatus* (Vahl) Woodson	Apocynaceae	100	15.5	3.29	0.5	1.93	3.14	2.78
*Pouteria venosa* (Mart.) Baehni	Sapotaceae	62	9.6	2.04	0.4	1.38	2.20	1.87
*Simaba orinocensis* Kunth	Simaroubaceae	30	4.7	0.99	0.8	3.06	1.51	1.85
*Pseudolmedia laevigata* Trécul	Moraceae	48	7.4	1.58	0.2	0.71	2.13	1.47
*Duroia eriopila* L.f.	Rubiaceae	47	7.3	1.55	0.1	0.46	1.88	1.30
*Astrocarium aculeatum* G.Mey	Arecaceae	37	5.7	1.22	0.2	0.76	1.63	1.20
*Pochota fendleri* (Seem) W.S. Alverson & M.C. Duarte	Malvaceae	9	1.4	0.30	0.7	2.59	0.38	1.09
*Guatteria schomburgkiana* Mart.	Annonaceae	30	4.7	0.99	0.2	0.74	1.51	1.08
*Duguetia lepdota* (Miq.) Pulle	Annonaceae	47	7.3	1.55	0.1	0.43	1.19	1.06
*Quiina rhytidopus* Tull.	Ochnaceae	26	4.0	0.86	0.1	0.21	1.51	0.86
Other 120	-	997	154.6	32.80	6.6	25.15	46.26	34.74
Total	43	3040	471.3	100	26.3	100	100	100

**Table 3.** Mean and standard deviation of structural parameters (stem diameter and total height) observed in ecotone forests of eastern Maracá Island, northern Brazilian Amazonia.

Classes (cm)	Palm			Tree		
	Height (m)	Diameter (cm)	Abundance (n)	Height (m)	Diameter (cm)	Abundance (n)
10-20	15.0±4.0	16.8±2.6	32	13.5±1.9	13.8±2.8	1598
20-30	15.4±5.3	25.4±2.8	138	19.2±1.4	23.8±3.0	591
30-40	19.7±5.7	32.1±1.7	55	23.3±1.0	34.0±2.9	297
40-50				26.2±0.8	43.9±3.0	159
> 50				29.8±2.0	62.5±13.1	170
Total	16.4±5.6	25.8±5.3	225	17.4±5.4	22.7±14.1	2815
