# Supplementary material for: Floristic composition in ecotone forests in northern Brazilian Amazonia: preliminary data
Source: Biodivers Data J. 2019 Oct 29;7:e47025. doi: 10.3897/BDJ.7.e47025 (PMC6831685; doi:10.3897/BDJ.7.e47025)
Supplement: Supplementary material 1 — The main studies in Roraima ecotone areas involving forest inventories and floristic surveys. [file bdj-07-e47025-s001.docx]

| **Studies/Projects** | **Study zones in Roraima** | **Reference** |
| --- | --- | --- |
| Projeto RADAMBRASIL | State of Roraima as a whole | Brazil-MME (1975) |
| Projeto RADAMBRASIL / floristic | State of Roraima as a whole | Moreira and Barbosa (2008) - floristic data from Brazil-MME (1975) |
| Projeto Maracá / floristic | Maracá Island | Milliken and Ratter (1998) |
| Projeto Maracá / monodominant forests | Maracá Island | Nascimento and Proctor (1997), Nascimento et al. (1997) |
| Campinas and campinaranas | central area of Roraima | Barbosa and Ferreira (2004) |
| Campinas and campinaranas | central area of Roraima | Gribel et al. (2008) |
| Campinas and campinaranas | central area of Roraima | Damasco et al. (2013) |
| Campinas and campinaranas / Dense Forests | central area of Roraima | Condé and Tonini (2013) |
| Forest fragments dispersed in savannas | savanna areas close Boa Vista city | Sette-Silva (1993) |
| Forest fragments dispersed in savannas | forest islands | Santos et al. (2013) |
| Forest fragments dispersed in savannas | forest islands | Jaramillo (2015) |
| Riverine forests | south region of Rio Branco basin | Alarcón and Peixoto (2007) |
| Riverine forests | south region of Rio Branco basin | Pereira (2018) |

**References**

Alarcón, J.G.S.; Peixoto, A.L. 2007. Florística e fitossociologia de um trecho de um hectare de floresta de terra firme, em Caracaraí, Roraima, Brasil. Boletim do Museu Paraense Emílio Goeldi (Ciências Naturais), 2(2): 33-60.

Barbosa, R.I.; Ferreira, C.A.C. 2004. Biomassa acima do solo de um ecossistema de “campina” em Roraima, norte da Amazônia Brasileira. Acta Amazonica, 34: 577–586.

Brazil-MME (1975). Projeto RADAMBRASIL - Levantamento de Recursos Naturais (volume 8). Ministério das Minas e Energia, Departamento Nacional de Produção Mineral, Rio de Janeiro. 426 p.  URL: <http://biblioteca.ibge.gov.br/visualizacao/livros/liv24025.pdf>

Condé, T.M.; Tonini, H. 2013. Fitossociologia de uma floresta ombrófila densa na Amazônia Setentrional, Roraima, Brasil. Acta Amazonica, 43(3): 247-260.

Damasco, Gabriel and Vicentini, Alberto and Castilho, Carolina V. and Pimentel, Tânia P. and Nascimento, Henrique E. M. 2013. Disentangling the role of edaphic variability, flooding regime and topography of Amazonian white-sand vegetation. Journal of Vegetation Science 24: 384--394. DOI: 10.1111/j.1654-1103.2012.01464.x

Gribel, R.; Ferreira, C.A.C.; Santos, J.L.; Coelho, L.S. 2008. Relatório preliminar da vegetação do Parque Nacional do Viruá – RR. Instituto Nacional de Pesquisas da Amazônia, Manaus, Amazonas. 46 pp.

Jaramillo, M.M.A. 2015. Estrutura, biomassa arbórea e composição florística de ilhas de mata da savana de Roraima, Norte da Amazônia Brasileira. Universidade Federal de Roraima, Boa Vista, Roraima, Brazil. 57 p.

Milliken, W.; Ratter, J.A. 1998. The vegetation of the Ilha de Maracá. In: Milliken, W.; Ratter, J.A. (Eds.). Maracá: the biodiversity and environment of an Amazonian rainforest. Wiley, Chichester, UK, p. 71-112.

Moreira, J.; Barbosa, R.I. 2008. Composição, riqueza e diversidade de árvores comerciais inventariadas pelo PROJETO RADAMBRASIL para Roraima e áreas adjacências. Mens Agitat, 3(2): 115-124.

Nascimento, M.T.; Proctor, J. 1997. Population dynamics of five tree species in a monodominant Peltogyne forest and two other forest types on Maracá Island, Roraima, Brazil. Forest Ecology and Management, 94(1-3): 115-128.

Nascimento, M.T.; Proctor, J.; Villela, D.M. 1997. Forest structure, floristic composition and soils of an Amazonian monodominant forest on Maracá Island, Roraima, Brazil. Edinburgh Journal of Botany, 54: 1-38.

Sette-Silva, E.L. 1993. Inventário preliminar das espécies arbóreas das florestas dos arredores de Boa Vista (Roraima) - uma abordagem fitossociológica. INPA/FUA, Manaus, AM. 194 p.

Santos, N.M.C.; Vale Júnior, J.F.; Barbosa, R.I. 2013. Florística e estrutura arbórea de ilhas de mata em áreas de savana do norte da Amazônia brasileira. Boletim do Museu Paraense Emílio Goeldi (Ciências Naturais), 8(2): 205-221.
